# Combined totally mini-invasive approach in necrotizing pancreatitis: a case report and systematic literature review

**DOI:** 10.1186/s13017-017-0126-5

**Published:** 2017-03-16

**Authors:** Luca Sorrentino, Osvaldo Chiara, Massimiliano Mutignani, Fabrizio Sammartano, Paolo Brioschi, Stefania Cimbanassi

**Affiliations:** 1 Trauma Team and Emergency Surgery, Niguarda Trauma Center, Niguarda Ca’ Granda Hospital, Piazza Ospedale Maggiore 3, Milan, 20162 Italy; 2 Digestive Endoscopy Service, Niguarda Ca’ Granda Hospital, Piazza Ospedale Maggiore 3, Milan, 20162 Italy; 3 Intensive Care Unit, Niguarda Trauma Center, Niguarda Ca’ Granda Hospital, Piazza Ospedale Maggiore 3, Milan, 20162 Italy

**Keywords:** Severe pancreatitis, Step-up approach, Video-assisted retroperitoneal debridement, Endoscopic transgastric necrosectomy, Percutaneous drainage, Walled-off pancreatic necrosis

## Abstract

**Background:**

Currently, both the step-up approach, combining percutaneous drainage (PD) and video-assisted retroperitoneal debridement (VARD), and endoscopic transgastric necrosectomy (ETN) are mini-invasive techniques for infected necrosis in severe acute pancreatitis. A combination of these approaches could maximize the management of necrotizing pancreatitis, conjugating the benefits from both the experiences. However, reporting of this combined strategy is anecdotal. This is the first reported case of severe necrotizing pancreatitis complicated by biliary fistula treated by a combination of ETN, PD, VARD, and endoscopic biliary stenting. Moreover, a systematic literature review of comparative studies on minimally invasive techniques in necrotizing pancreatitis has been provided.

**Case presentation:**

A 59-year-old patient was referred to our center for acute necrotizing pancreatitis associated with multi-organ failure. No invasive procedures were attempted in the first month from the onset: enteral feeding by a naso-duodenal tube was started, and antibiotics were administered to control sepsis. After 4 weeks, CT scans showed a central walled-off pancreatic necrosis (WOPN) of pancreatic head communicating bilateral retroperitoneal collections. ETN was performed, and bile leakage was found at the right margin of the WOPN. Endoscopic retrograde cholangiopancreatography confirmed the presence of a choledocal fistula within the WOPN, and a biliary stent was placed. An ultrasound-guided PD was performed on the left retroperitoneal collection. Due to the subsequent repeated onset of septic shocks and the evidence of size increase of the right retroperitoneal collection, a VARD was decided. The CT scans documented the resolution of all the collections, and the patient promptly recovered from sepsis. After 6 months, the patient is in good clinical condition.

**Conclusions:**

No mini-invasive technique has demonstrated significantly better outcomes over the others, and each technique has specific indications, advantages, and pitfalls. Indeed, ETN could be suitable for central WOPNs, while VARD or PD could be suggested for lateral collections. A combination of different approaches is feasible and could significantly optimize the clinical management in critically ill patients affected by complicated necrotizing pancreatitis.

## Background

Severe necrotizing pancreatitis is related to a high mortality rate, ranging from 20% in patients with sterile necrosis up to 40% in case of infected necrosis associated with multi-organ failure (MOF) [1, 2]. Therefore, occurrence of severe sepsis doubles the risk of death, and this mortality is even higher with increasing age [2, 3]. Early open surgery has been initially proposed for necrotizing pancreatitis, but poor outcomes were observed due to the high risk of bleeding and pancreatic or colonic fistula, leading to a perioperative morbidity of 50–60% and a mortality rate equal to 20–25% [4, 5]. Since early open surgery could worsen prognosis, nowadays, other less invasive procedures, such as percutaneous drainage (PD), endoscopic transgastric necrosectomy (ETN), or video-assisted retroperitoneal debridement (VARD) are suggested [5, 6]. Therefore, open surgical necrosectomy with repeated laparotomies is considered the last choice whereas other therapeutic options have failed. Currently, the step-up approach, which includes PD possibly followed by VARD or endoscopic transluminal drainage followed by ETN, is proposed as a standard of care for necrotizing pancreatitis [6]. However, few trials have compared the step-up approach with open necrosectomy; therefore, a consensus on the best timing and management of these techniques is lacking [6, 7]. Moreover, the percutaneous step-up approach is certainly useful for lateral fluid or necrotic collections, but its role for medial collections (such as those posteriorly to the stomach) is much more controversial, and ETN could be more suitable in these cases [6]. Combined approaches could conjugate benefits from both endoscopic and percutaneous or minimally invasive drainages, thus representing a possible solution for severe necrotizing pancreatitis, but they are anecdotal and rarely reported in literature above all in case of pancreatitis-related complications [8]. Particularly, biliary fistula involving the common bile duct is a rare complication of acute necrotizing pancreatitis, its pathogenesis is supposed to be related to the necrotizing inflammatory process and its management is considered to be extremely difficult [9]. Here, we present the first case in our knowledge of a patient affected by severe necrotizing pancreatitis complicated by biliary fistula, successfully treated by a combined minimally invasive approach conjugating ETN, PD, VARD, and endoscopic retrograde cholangiopancreatography (ERCP) with biliary stenting.

## Case presentation

A 59-year-old man was admitted to a community hospital for worsening abdominal pain in the upper quadrants with jaundice (total bilirubin 13.4 mg/dL) and evidence of highly elevated serum amylase (5400 U/L). An abdominal ultrasound showed cholelithiasis with common bile duct dilatation due to biliary sludge. Due to the presence of biliary obstruction, an ERCP was attempted but failed due to impossible cannulation of the papilla; therefore, the procedure was immediately interrupted. Moreover, 2 days after patient hospitalization in the primary hospital, his clinical conditions worsened by the development of MOF, since acute respiratory distress syndrome (ARDS) and acute renal failure with anuria occurred. Therefore, the patient underwent endotracheal intubation and was referred to the intensive care unit of our referral tertiary center. The patient had no relevant past history or medications. On admission at our hospital, the patient presented with elevated serum creatinine (4.26 mg/dL, normal range 0.7–1.2 mg/dL) and blood urea nitrogen (111 mg/dL, normal range 18–48 mg/dL). The serum amylase was equal to 233 U/L (normal range 28–100 U/L), and the total bilirubin was 8.33 mg/dL (normal range 0.25–1 mg/dL). A marked anemia was evident (hemoglobin 7.8 g/dL, hematocrit 23.6%) without leukocytosis. Arterial blood gas analysis revealed hypoxia without acidosis (pH 7.41, base excess −0.2, lactate 1 mmol/L). The APACHE II score at admission was 30. Enteral feeding was started immediately upon naso-duodenal tube placement, and continuous veno-venous hemofiltration (CVVH) was introduced for acute renal failure also considering the occurrence of hyperkalemia up to 6 mmol/L. Continuous vasopressor support was not necessary, as hemodynamic stability was maintained by fluid infusion.

However, several episodes of shock associated with hyperpyrexia up to 38.5 °C occurred and required short-course treatment with vasopressor. These episodes were hemodynamically consistent with septic shock, but blood cultures were initially negative, as well as skin and mucosal swabs. Only urine cultures were found positive for *Escherichia coli*; therefore, piperacillin/tazobactam therapy was started based on antibiograms. Due to persistent ARDS with PaO_2_/FiO_2_ ratio <100 mmHg, the patient needed prolonged artificial ventilation, and a percutaneous tracheostomy was performed. Several attempts of weaning from mechanical ventilation failed. Intra-abdominal pressure was monitored, being constantly equal to 12 mmHg. Abdominal ultrasound confirmed the presence of distended gallbladder with multiple gallstones and biliary sludge without signs of acute cholecystitis, but common bile duct diameter was normal, apparently with no more evidence of obstructing sludge into the biliary tree.

**Fig. 1:**
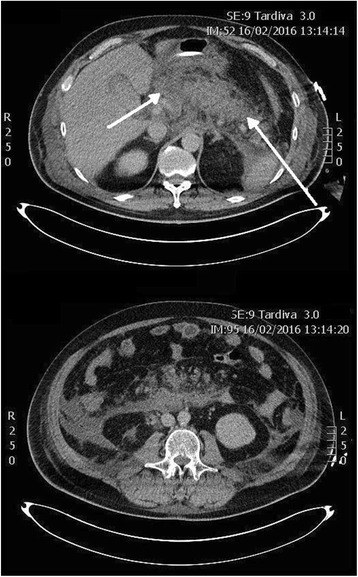
First CT scans at admission. Acute pancreatic necrosis and edema of pancreatic tail and surrounding adipose tissue are visible (*arrows*)

The patient underwent CT scan which showed acute necrosis of the cephalic portion of the pancreas, with reduced enhancement in pancreatic tail and a 11 × 5.5 cm fluid collection in the right retroperitoneal space (Fig. 1). Severe edema of peri-pancreatic adipose tissue was documented, with abdominal and pelvic effusion. Urine iodine excretion was negligible, and bilateral pleural effusion was documented. Weekly follow-up CT scans demonstrated the appearance of a 13 × 9 cm highly dense retroperitoneal collection cranial to the pancreatic head communicating with another 6 × 5 cm collection near the left hepatic lobe. Head and neck of the pancreas were no more recognizable, and a significant compression was evident on portal vein. Moreover, a progressive increase in bilateral retroperitoneal fluid collections was detected, as well as a further 12 × 4 cm necrotic collection under the uncinate process.

**Fig. 2:**
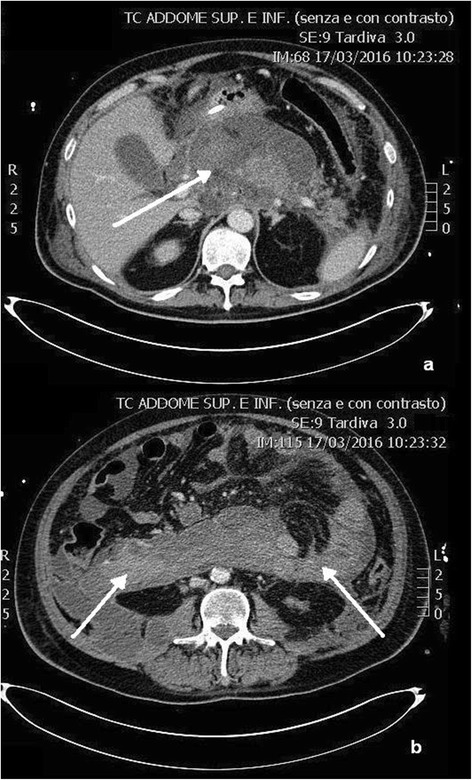
CT scans after 4 weeks. Acute pancreatic necrosis is replaced by a central walled-off pancreatic necrosis (**a**, *arrow*) and lateral retroperitoneal fluid collections (**b**, *arrows*)

**Fig. 3:**
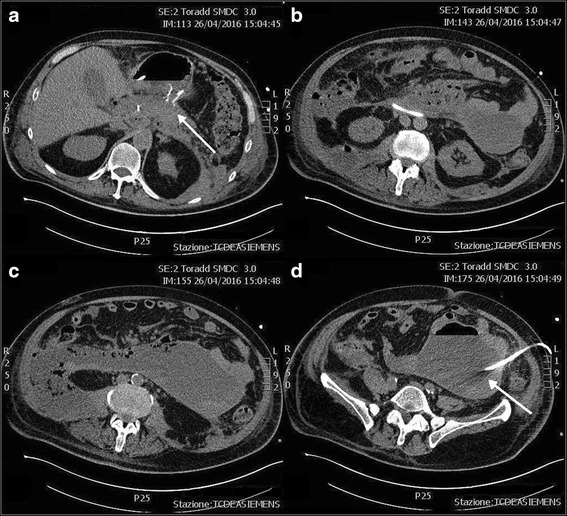
**a**–**d** Transgastric necrosectomy and percutaneous drainage. The central necrotic collection has been almost completely solved after transgastric necrosectomy (**a**, *arrow*), and a percutaneous drainage was performed on the left retroperitoneal collection (**d**, *arrow*)

Four weeks after the admission, these collections were in communication with a unique large 24 × 10 cm retroperitoneal collection characterized by a partially fluid content. Also, the necrotic collection cranial to the pancreatic head connected with the left hepatic lobe collection augmented, with a diameter of 15 × cm, and acquired features of walled-off pancreatic necrosis (WOPN) (Fig. 2). Due to the worsening of retro-gastric WOPN with gastroduodenal extrinsic compression and the clinical evidence of ongoing sepsis, the patient was subjected to ETN of the WOPN. A 1.6 × 2 cm metallic stent (Niti-S yo-yo stent, Taewoong, South Korea) was positioned, draining abundant necrotic and purulent material, and a 6-Fr naso-cystic tube was then positioned to guarantee continuous irrigation of the drained WOPN with 2 L saline/die (Fig. 3a). Cultures on purulent material revealed the presence of *Stenotrophomonas maltophilia* and blood cultures were positive for *E. coli*; therefore, antibiotics cefotaxime and sulfamethoxazole/trimethoprim were started targeted on antibiograms.

**Fig. 4:**
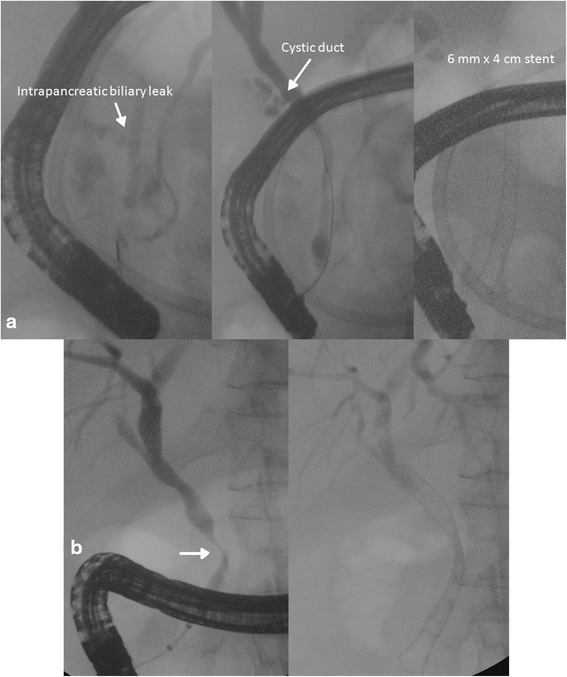
ERCP and biliary stenting. Cholangiography confirmed an intrapancreatic biliary leak (*arrow*), and a 18 Fr × 4 cm biliary stent was positioned (**a**). ERCP after patient discharge demonstrated a common bile duct post-inflammatory stenosis (*arrow*), and a 24 Fr × 6 cm biliary stent was positioned (**b**)

Endoscopic transgastric pancreatic necrosectomy was repeated two times over the following 2 weeks, and during the last endoscopy, a biliary leakage was encountered from an unspecified site on the right margin of the WOPN. Subsequently, the patient repeated abdominal CT scan which confirmed a significant reduction of the WOPN, being 5.6 × 4.3 cm, while the large right and left retroperitoneal collections increased in size (Fig. 3b, c). Then, an ultrasound-guided PD was performed of the left retroperitoneal collection, positioning a 10-Fr drainage tube (Fig. 3d). Due to size reduction of the WOPN and consequent less extrinsic compression on the duodenum, an ERCP became feasible. ERCP confirmed a biliary fistula from the distal third of the common bile duct to the WOPN cavity; therefore, sphincterotomy of papilla was performed and a 6 mm × 4 cm fully covered metallic biliary stent (Wallflex, Boston Scientific, Boston, MA, USA) was positioned (Fig. 4a). Endoscopic second-look documented almost complete debridement of the WOPN.

**Fig. 5:**
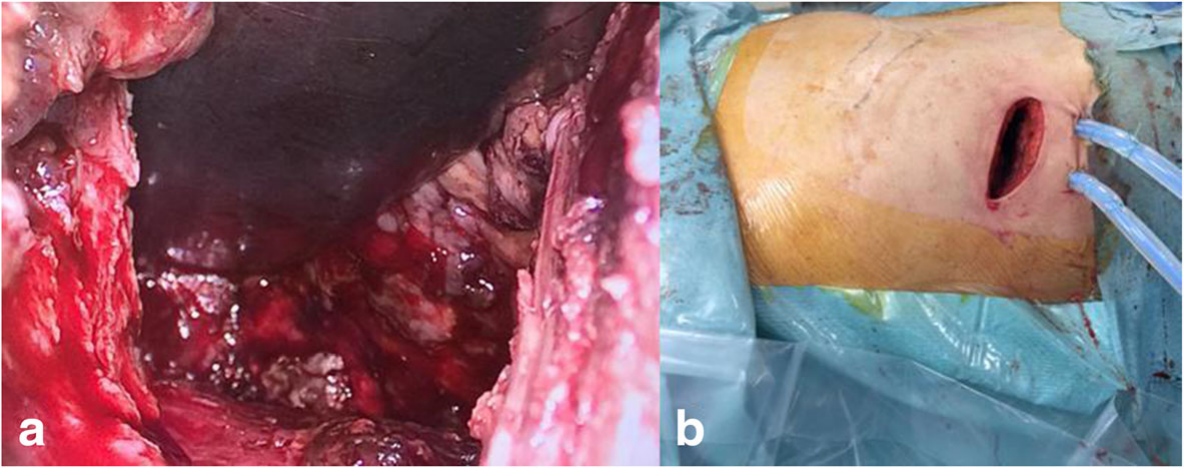
Video-assisted retroperitoneal debridement. VARD was performed on the *right* retroperitoneal necrotic collection (**a**). Two large bore drains were *left* for lavage and drainage of the necrotic cavity (**b**)

The following CT scans showed a progressive reduction of both the WOPN and the left retroperitoneal collection but only a modest decrease of the large right retroperitoneal necrotic collection which appeared only partially liquefied. Moreover, the patient remained febrile despite meropenem, colistin, and fluconazole were started after *Klebsiella pneumoniae* carbapenemase-producing bacteria and *Candida albicans* were isolated on pus collected from abdominal drainage and on blood cultures. Therefore, VARD was decided 2 weeks after the last ETN. The patient was positioned on the left flank; the right lower costal margin and the iliac crest were marked on the skin. Ultrasound was used to localize the necrotic retroperitoneal tissue underlying the skin between markings and a 5-cm incision was performed on this site, about 2 cm above the iliac crest. Once the access to the retroperitoneal space is completed, necrotic material was evacuated by aspiration. Then, a 10-mm laparoscope was inserted through the incision without gas insufflation, and the right retroperitoneal space was explored. Debridement of necrotic material was performed by forceps and with a laparoscopic jet irrigation/suction device, and a plentiful quantity of infected debris was evacuated. When the descending part of the duodenum was visualized frontally, with the inferior vena cava being visible inferiorly and the right mesocolon superiorly, the VARD was considered completed (Fig. 5a). The retroperitoneal space was then flushed with 4 L saline solution, and two 32-Fr drainages were positioned superiorly and inferiorly; the fascia and skin were closed (Fig. 5b). The procedure was uneventful, and no bleeding occurred intraoperatively or in the post-operative course. A continuous irrigation with 5 L saline solution was infused in 24 h via the superior drainage, and fluids were collected by the inferior drainage.

**Fig. 6:**
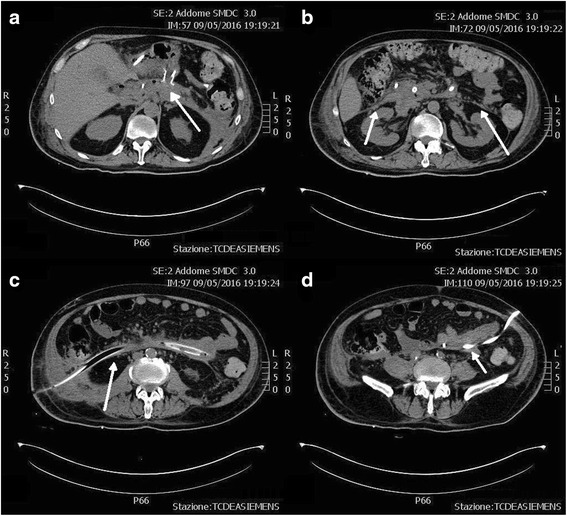
**a**–**d** CT scans after VARD. After transgastric necrosectomy, VARD, and percutaneous drainage, all the retroperitoneal necrotic collections dramatically reduced (*arrows*)

Subsequently, a gradual decrease of inflammatory markers was recorded together with an improvement of clinical conditions, and the patient became afebrile after 6 days from the VARD. The following CT scan demonstrated a significant reduction of all abdominal collections, being the right retroperitoneal collection sized 7.5 × 4 cm (Fig. 6). No biliary material was detected in drainages. Two weeks after VARD, all antibiotics were stopped, creatinine and serum electrolytes were in normal ranges, and CVVH was terminated. The patient was completely weaned from lung ventilation and fluid challenge, and tracheostomy was closed. Enteral feeding from naso-duodenal tube was continued; the patient was discharged from the intensive care unit and transferred to the surgical ward. Subsequent CT scan showed further reduction of all intra-abdominal collections and the left drainage was removed, while only one right drainage positioned after VARD was left and continued to drain purulent material. The patient gradually started per os feeding and enteral feeding stopped. After 3 weeks, CT scan was repeated and documented reduction of the right retroperitoneal collection and the large right drainage was therefore replaced with a 10-Fr drainage which was removed after 2 weeks. The patient was discharged in good clinical conditions, and 1 month after discharge, a CT scan demonstrated resolution of the intra-abdominal collections. A subsequent ERCP was performed to remove the biliary stent but a common bile duct post-inflammatory stenosis was detected; therefore, a new 8 mm × 6 cm fully covered metallic biliary stent (Wallflex, Boston Scientific, Boston, MA, USA) was positioned (Fig. 4b). After 2 months, the transgastric metallic stent used for ETN and biliary stent were both removed, with no residual structure of the bile duct.

## Literature review

A systematic review was performed by searching in PubMed the following keywords: “necrotizing pancreatitis” AND “necrosectomy” OR “VARD” OR “percutaneous drainage” OR “walled-off pancreatic necrosis”. Only comparative studies or randomized clinical trials about interventions for necrotizing pancreatitis, from 2000 to 2017, were included. Sixty-eight studies were found and analyzed: 11 were excluded due to a single-arm design without a control group, 28 were excluded because they were not relevant or off-topic, 2 were excluded because outcomes were not properly reported, and 10 were excluded because they were not in English language. Therefore, a total of 18 studies were included in the systematic review (Table 1).

**Table 1:** Main studies assessing minimally invasive techniques for severe necrotizing pancreatitis

Study	Design	Number of cases	Outcomes	Limitations
van Santvoort et al. [7]	Open necrosectomy vs. PD ± VARD, randomized controlled trial	88 patients (45 open necrosectomy vs. 43 PD ± VARD)	Major complications occurred in 69% of open necrosectomy patients vs. 40% of PD ± VARD patients (*p* = 0.006); open necrosectomy was associated with more incisional hernias (*p* = 0.03) and new-onset diabetes (*p* = 0.02). Mortality was not different.	Trial not designed to assess differences in mortality.
Bakker et al. [22]	ETN vs. VARD or open necrosectomy, randomized controlled trial	20 patients (10 ETN vs. 10 VARD/open necrosectomy)	ETN reduced both the proinflammatory response (*p* < 0.004) and major complications, multiple organ failure or death (*p* = 0.03)	Small number of patients
Bausch et al. [23]	VARD vs. ETN vs. open necrosectomy, retrospective study	32 patients (14 VARD vs. 18 ETN vs. 30 open necrosectomy)	Open necrosectomy had higher overall mortality (*p* < 0.05), ongoing sepsis rates and bleedings; ETN was complicated by gastric perforation in 28% of cases requiring immediate laparotomy	Small number of patients, retrospective study.
van Brunschot et al. [24]	Endoscopic transgastric drainage ± ETN vs. PD ± VARD, randomized controlled trial	98 patients	End-points are rates of major complications, need for re-interventions, quality of life, and cost-analysis between endoscopic step-up approach and surgical step-up approach. Results are still awaited.	Results still awaited
Kumar et al. [19]	ETN vs. PD ± open necrosectomy, matched cohort study	24 patients (12 ETN vs. 12 PD ± open necrosectomy)	ETN was superior in clinical remission rate (*p* < 0.01) and reduced major complications, length of stay, and post-operative health care utilization (*p* < 0.01).	Small number of patients.
Rasch et al. [25]	Open necrosectomy vs. endoscopic transgastric drainage ± ETN ± PD, retrospective multicenter study	220 patients (30 open necrosectomy vs. 190 step-up approach)	Lower complication rate (44.7 vs. 73.3%, *p* < 0.001), lower mortality (10.5 vs. 33.3%, *p* = 0.002), and lower incidence of diabetes (4.7 vs. 33.3%, *p* < 0.001) was demonstrated with step-up approach compared to open necrosectomy; 18.9% of step-up approach patients required open necrosectomy.	Retrospective study
Carter et al.[10]	Open necrosectomy + percutaneous necrosectomy vs. upfront percutaneous necrosectomy, case series	14 patients (4 open necrosectomy vs. 10 percutaneous necrosectomy)	Upfront percutaneous necrosectomy by sinus tract endoscopy had 20% mortality vs. 0% with open necrosectomy, but only 40% of patients required ICU (vs. 100%).	Retrospective case series, small number of patients
Gardner et al. [18]	Endoscopic transgastric drainage vs. ETN, retrospective study	45 patients (25 ETN vs. 20 endoscopic transgastric drainage)	Walled-off pancreatic necrosis successfully resolved in 88% of patients treated with ETN vs. 45% with endoscopic drainage (*p* < 0.01).	Retrospective study, referral center bias
Raraty et al. [12]	Open necrosectomy vs. VARD, retrospective study	189 patients (52 open necrosectomy vs. 137 VARD)	Organ failure in 31% of patients treated by VARD vs. 56% with open necrosectomy (*p* < 0.0001); 43 vs. 77% respectively required ICU support. Mortality rate was 19% with VARD vs. 38% with open necrosectomy (*p* = 0.009).	Retrospective study, referral center bias
Guo et al. [14]	Open necrosectomy vs. retroperitoneal necrosectomy, retrospective study	412 patients (108 retroperitoneal necrosectomy vs. 304 open necrosectomy)	Mortality rate was 8.3% with retroperitoneal necrosectomy vs. 20.4% with open necrosectomy (*p* = 0.004); complications rate and mean ICU stay were significantly lower.	Retrospective study
Tan et al. [20]	Open necrosectomy vs. ETN, multicentric retrospective study	32 patients (21 open necrosectomy vs. 11 ETN)	Acute complications rate was 86% in open necrosectomy vs. 27% with ETN (*p* = 0.002), ICU stay and hospitalization were significantly reduced with ETN.	Clinical severity scores were unbalanced between groups, retrospective study, small number of patients
Bang et al. [26]	Endoscopic transgastric drainage ± ETN vs. “algorithmic approach” including endoscopic drainage, ETN, PD, percutaneous necrosectomy, open necrosectomy, observational study	100 patients (47 endoscopic transgastric drainage ± ETN vs. 53 “algorithmic approach”)	Treatment success rate equal to 91% with “algorithmic approach” vs. 60% with endoscopic drainage ± ETN (*p* < 0.001).	Observational study without randomization, unbalanced gender and race between groups
van Santvoort et al. [11]	Open necrosectomy vs. VARD, case-matched retrospective study	30 patients (15 open necrosectomy vs. 15 VARD)	Post-operative multiple organ failure occurred in 2 patients in VARD group vs. 10 patients treated by open necrosectomy (*p* = 0.0008). Complications and mortality rates were equal between groups.	Retrospective study, small number of patients
Senthil Kumar et al. [15]	Open necrosectomy vs. VARD, case-matched retrospective study	30 patients (15 open necrosectomy vs. 15 VARD)	Post-operative complications in 26.6% of patients treated by VARD vs. 53.3% of patients treated by open necrosectomy (*p* = 0.248). Re-interventions, ICU stay, and hospitalization were similar.	Retrospective study, small number of patients
Pupelis et al. [16]	Open necrosectomy vs. ultrasound-focused necrosectomy, prospective study	58 patients (36 necrosectomy vs. 22 focused necrosectomy)	Resolution of sepsis was earlier with focused necrosectomy; ICU stay longer with open necrosectomy (*p* = 0.024).	Mini-invasive transgastric or percutaneous techniques not considered.
Tu et al. [13]	Open necrosectomy vs. VARD, retrospective study	50 patients (32 open necrosectomy vs. 18 VARD)	VARD was associated with shorter operative time (130 vs. 148 min, *p* = 0.007) and shorter hospitalization (40.8 vs. 55.9 days, *p* = 0.053) compared to open necrosectomy. Fewer complications with VARD (43.8 vs. 27.8%).	Retrospective study
Gluck et al. [17]	Endoscopic transgastric drainage + PD vs. PD only, retrospective study	95 patients (49 endoscopic drainage + PD vs. 46 PD only)	Endoscopic drainage + PD significantly reduced hospitalization, CT scans, and ERCPs (*p* < 0.05) compared to PD only.	Retrospective study
Woo et al. [21]	Endoscopic transgastric drainage or ETN vs. open necrosectomy vs. PD	30 patients (12 endoscopic treatment vs. 8 PD vs. 10 open necrosectomy)	Mean hospitalization time was 62 days with endoscopic treatment vs. 101 days with PD and 91 days with open necrosectomy (*p* = 0.046). Pancreatic fistula and new-onsetdiabetes were more frequent with open necrosectomy (*p* = 0.04, *p* = 0.012).	Retrospective study, small number of patients

*PD*, percutaneous drainage, *ETN* endoscopic transgastric necrosectomy, *VARD* video-assisted retroperitoneal debridement

Since the open necrosectomy with lateral approach proposed by Fagniez for peri-pancreatic debridement through the retrocolic space, various mini-invasive techniques have been described to replace such a complicated surgery [4, 6]. Carter et al. initially described a novel technique of mini-invasive percutaneous necrosectomy by positioning under CT guidance an 8-F nephrostomy catheter into the necrotic cavity, by passing between the spleen and the splenic flexure of the colon on the left side, or through the gastrocolic omentum on the right side [10]. The path traced by this catheter was therefore gradually dilated by the surgeon to insert a 30-F Amplatz, and necrosectomy was finally performed with the aid of an operative nephroscope. A post-operative continuous lavage was then allowed through positioned drains. In case of need to repeat necrosectomy, this was performed by sinus tract endoscopy using a flexible or rigid endoscope through the precedent percutaneous path, with the aid of endoscopic snares or forceps. Percutaneous necrosectomy performed after prior open surgery was compared to percutaneous necrosectomy as a first-line treatment in a comparative study on 14 patients affected by necrotizing pancreatitis: interestingly, in the latter group, open surgery was safely avoided in 80% of patients who were discharged after a median of 3 percutaneous procedures only [10]. Moreover, only 40% of these patients required post-operative intensive care unit (ICU). However, 20% mortality was reported with upfront percutaneous necrosectomy vs. 0% in patients previously treated with open necrosectomy.

After that initial experience, subsequent studies have systematically compared open necrosectomy to percutaneous necrosectomy or VARD, to assess if these minimally invasive retroperitoneal approaches were associated with better outcomes in terms of complications, mortality, and length of stay. van Santvoort and the Dutch Acute Pancreatitis Study Group have published in 2007 a comparative study on 30 patients with infected necrotizing pancreatitis: 15 patients who were treated by VARD were case-matched and compared to 15 patients subjected to standard open necrosectomy by laparotomy [11]. No significant differences were observed both in post-operative complications requiring re-intervention (*p* = 1.000) and in mortality rate (*p* = 0.08), but new-onset MOF occurred more frequently in the open necrosectomy group (*p* = 0.008). These results were encouraging and advocated the need of a randomized controlled trial on this topic.

Three years later, the same research group published the first randomized clinical trial on open necrosectomy vs. step-up approach with PD followed by VARD if necessary in a cohort of 88 patients affected by necrotizing pancreatitis, who were randomly assigned to the two treatment arms [7]. A significant reduction of new-onset MOF was confirmed with the step-up approach (12 vs. 40% with open necrosectomy, *p* = 0.002), as well as incisional hernias (7 vs. 24%, *p* = 0.03) and new-onset diabetes (16 vs. 38%, *p* = 0.02). Interestingly, major complications including visceral perforation, entero-cutaneous or pancreatic fistula, and bleeding occurred in 40% of patients treated by the step-up approach vs. 69% of patients treated by open necrosectomy (*p* = 0.006). However, mortality was equal between groups and the study was not designed to assess a difference in mortality. Notably, up to 35% of patients in the step-up approach group were successfully treated with PD only without the need of subsequent VARD, suggesting that aggressive necrosectomy could be spared in favor of simple drainage of infected fluid in a substantial proportion of patients.

According to these findings, a large retrospective study on 189 patients treated in a tertiary referral center demonstrated a significant benefit of VARD over open necrosectomy in terms of needed ICU support (*p* < 0.0001) and complications (55 vs. 81%, *p* = 0.001) [12]. Moreover, this study demonstrated also a significant reduction in mortality with VARD (19 vs. 38%, *p* = 0.016), being the use of a minimally invasive necrosectomy an independent predictor of mortality together with age and preoperative status of MOF. Other advantages associated with VARD and globally to retroperitoneal approaches compared to open necrosectomy with anterior laparotomy were shorter operative times and shorter hospitalization in two more recent large-population retrospective study [13, 14]. Conversely to these evidence, Senthil Kumar et al. reported that post-operative complications were fewer but not significantly different with VARD compared to open necrosectomy, being re-intervention rate, ICU stay, and overall hospital stay similar between groups [15]. This study was case-matched, but its retrospective design and the small size of cohort could explain those controversial findings.

Interestingly, retroperitoneal debridement could be further improved by ultrasound-guided navigation and positioning of percutaneous catheters. Pupelis et al. demonstrated an earlier resolution of sepsis and a shorter ICU stay with ultrasound-guided focused necrosectomy compared to conventional open necrosectomy in a cohort of 58 patients [16]. Minimally invasive retroperitoneal necrosectomy and PD are particularly suitable in case of lateral necrotic collections extended in the retroperitoneal gutters, but central WOPN are much less manageable with these approaches. Endoscopic transgastric routes could represent a better indication for such collections. Recently, Gluck et al. have retrospectively evaluated the clinical impact of adding endoscopic transgastric drainage to PD compared to PD alone on 95 cases of necrotizing pancreatitis [17]. Endoscopic drainage led to shorter length of stay, fewer CT scans, and reduced use of ERCP (*p* < 0.05), although mortality did not vary between cohorts.

However, endoscopic drainage can easily resolve fluid collections, while it could be less effective for mostly solid WOPN. Indeed, in 2009, Gardner and colleagues investigated the use of endoscopic transgastric drainage vs. ETN and found that WOPN successfully resolved in 88% of patients treated by ETN vs. 45% of patients treated by endoscopic drainage only, with the same number of procedures (*p* < 0.01) [18]. Subsequently, two studies have explored the clinical benefit of ETN vs. PD or open necrosectomy: ETN was found to be superior in clinical remission rate, reduced complications, shorter hospitalization, and ICU stay [19, 20]. In particular, major complications were recorded in 86% of open necrosectomy patients vs. 27% only in ETN patients [20]. More recently, Woo et al. retrospectively compared ETN, PD, and open necrosectomy on 30 patients: the mean hospitalization time was 62 days with ETN, 91 days with open necrosectomy, and 101 days with PD (*p* = 0.046), but pancreatic fistula and new-onset diabetes were more frequent with open necrosectomy [21].

Then, literature review demonstrated that both retroperitoneal and endoscopic transgastric mini-invasive techniques are associated with less complications compared to open necrosectomy in management of infected or unsolving or symptomatic WOPN. However, few clinical trials have compared the alternative use of these minimally invasive procedures, and fewer studies have investigated and reported their combination. The PENGUIN trial investigated the use of ETN in 10 patients compared to open necrosectomy or VARD in 10 patients and demonstrated superiority of the former technique in terms of reduced global morbidity and mortality rate, from 80 to 20% [22]. These results should be prudently considered, but certainly, a noninferiority of ETN versus VARD could be inferred. Conversely, a retrospective study on 62 patients comparing open necrosectomy vs. VARD and vs. ETN showed a significant reduction both in complications and in mortality with the mini-invasive approaches compared to open necrosectomy [23]. Notably, both VARD and ETN were superior to open surgery, but the two mini-invasive techniques themselves had almost equivalent outcomes. Interestingly, the rate of re-laparotomy was higher in the ETN group because of free gastric perforation, a specific complication of the technique which occurred in 28% of cases, particularly when a proper transgastric window for the procedure with a large contact between gastric wall and WOPN is not present.

More recently, the TENSION trial has been designed to compare the surgical step-up approach (PD followed by VARD) versus the endoscopic step-up approach (endoscopic transgastric drainage followed by ETN); results of the TENSION trial are intensely expected [24]. However, again the two pathways have been considered as separate alternatives and not as possibly combined procedures. Conjugation of the two techniques has been recently reported in literature for the first time on a patient with severe acute pancreatitis complicated by infected WOPN extended laterally to the retroperitoneal spaces, similarly to our patient [8]. In this case report, the WOPN was complicated by multiple enteric fistulae, and endoscopic transgastric drainage was necessarily completed by VARD for a proper control of retroperitoneal collections, suggesting that a mini-invasive approach combining all the abovementioned techniques could maximize the clinical benefit in the critically ill patient [8].

Indeed, as previously stated, ETN is preferable for management of pancreatic or peri-pancreatic WOPN, while lateral collections are preferentially treated by PD or VARD based on the proportion of fluid and solid necrosis. Moreover, lateral collections could spread not only from a medial WOPN as a result of necrosis extension but also if retroperitoneal compartmentalization is lost after transgastric necrosectomy and lavage through a naso-cystic tube, with subsequent spread of necrotic fluids in communicating recesses and cavities. In these cases, PD for fluid collections and VARD for more solid collections may be taken in consideration.

A large retrospective study has compared open necrosectomy vs. a step-up approach consisting in transgastric drainage and/or ETN and/or PD in 220 patients affected by severe necrotizing pancreatitis [25]. Interestingly, this study assessed the usefulness of combination of transgastric and percutaneous routes to control infected necrosis, although the use of VARD was not reported. The study showed a markedly reduced complications rate with the step-up approach (44.7 vs. 73.3%, *p* < 0.001), together with lower mortality (10.5 vs. 33.3%, *p* = 0.002) and lower incidence of diabetes (4.7 vs. 33.3%, *p* < 0.001) compared to open necrosectomy. However, 18.9% of step-up approach patients later required open necrosectomy [25].

Finally, an observational study without randomization on 100 patients has compared upfront endoscopic transgastric drainage with or without ETN vs. a so-called algorithmic approach which consisted of a stepwise combined approach based on size and location of WOPN [26]. If WOPN was central and <12 cm in diameter, endoscopic transgastric drainage was performed as a first-line treatment. In case of WOPN >12 cm or extended to the retroperitoneal gutters or not responding after endoscopic transgastric drainage, ETN was performed by creating multiple transgastric conduits (multiple transluminal gateway technique). Suboptimal response after four procedures was probably related to the prevalent lateral extension of WOPN into the retroperitoneum; therefore, a percutaneous sinus tract endoscopic necrosectomy was subsequently performed. In case of failure of the abovementioned techniques, open necrosectomy was considered. Interestingly, treatment success rate was significantly higher for “algorithmic approach” compared to upfront endoscopic drainage or ETN (91 vs. 60%, *p* < 0.001) [26]. Moreover, using the algorithmic approach was the only independent predictor of successful treatment, although open necrosectomy was included among treatments. This study was not designed as a randomized trial and VARD was not used; therefore, its findings should be cautiously considered. However, it suggests that even open necrosectomy could still be a proper indication as a last chance in selected patients, if a wise and thoughtful combination of minimally invasive approaches is used. The key for success for WOPN management could rely in a proper timing of multiple adequate treatments, rather than in type of treatment itself.

## Discussion

The step-up approach is becoming a treatment option for necrotizing acute pancreatitis instead of early surgery. Its philosophy is expressed by the “3D” concept: delay, drain, and debride [27]. It means that the optimal management should include a first step of intensive care treatment for recovery from challenging clinical conditions, waiting the formation of WOPN. Generally, this step should last 4 weeks at least, when a well-defined walled-off necrosis or pseudocyst is expected. Then, a second step for percutaneous or endoscopic drainage should follow, eventually completed by the third step which consists of minimally invasive surgery if debridement is needed [6, 27].

In the presented case, 6 weeks were necessary before a well-defined, although not capsulated, necrotic collection near the pancreatic head was visible. Since the worsening of clinical conditions notwithstanding maximal intensive care support, an ETN was first attempted for central WOPN, while PD was preferred on the left retroperitoneal collection since it was mostly fluid and in a lateral position, easily approachable by ultrasound exploiting the lumbar window. However, despite a significant reduction in size of these infected collections after repeated ETN with a proper antibiotic therapy, sepsis control was still not achieved and the right retroperitoneal collection progressively increased. The VARD was chosen instead of PD because of the semi-solid nature of the right collection. VARD allowed a prompt and extensive debridement of most of the right retroperitoneal infected necrosis, with a fast resolution of severe sepsis.

Most of the abovementioned studies have demonstrated a better clinical outcome with minimally invasive techniques compared to open necrosectomy. However, due to the heterogeneity of these studies and between each case of necrotizing pancreatitis, definitive evidence-based conclusions about the best surgical approach are currently difficult to be drawn. Only few randomized clinical trials are available on this topic, but these evidence could be biased by small number of patients or a study design not suitable to assess differences in mortality, which should be a main endpoint. Several other studies were based on retrospectively reviewed cohorts of patients treated in different centers or in the same hospital but by different surgeons with different approaches and different timings. Therefore, the differences in outcomes between open necrosectomy and minimally invasive techniques could be also explained by the delayed time often occurred before the use of ETN or PD or VARD compared to open necrosectomy and not only by significant morbidity associated with surgical necrosectomy itself [28]. Moreover, other variables could have impacted on outcomes, such as the preferential use of early enteral feeding or the possible clinical stabilization in ICU before any invasive procedure. This point was endorsed by the recent work by Bang and colleagues, which demonstrated that a step-by-step approach even including open necrosectomy could be associated with a significantly higher success rate than endoscopic debridement as a stand-alone therapy [26]. These data suggest that probably, it is not the specific technique itself to determine the outcome, but a wise multi-staged combined approach with adequate timing, possibly including surgical necrosectomy if indicated.

Furthermore, minimally invasive techniques are not free from severe complications. Severe hemorrhage is widely reported, occurring in up to 20% of patients after VARD [7, 23]. A mortality rate up to 30–40% is observed in case of procedure-related hemorrhage, being this risk particularly high in early procedures due to the intensively vascularized inflammatory collections and the frequent impairment in coagulation [6]. Therefore, a hybrid room should be available 24/24 for emergency hemorrhage control, both intravascular and/or surgical, in case of life-threatening bleeding.

Also, colonic perforation is reported as a complication of VARD in 15% of patients, but it is unusual if necrosectomy is avoided in the first weeks, thanks to the easier feasibility after procedure delay [29, 30]. Therefore, avoiding necrosectomy in the first weeks after necrotizing pancreatitis facilitates procedures on necrotic collections and improves the outcome, as what occurred in our case.

A specific complication of ETN could be gastric perforation. In the trial by Bausch et al., ETN was associated with gastric perforation in about one third of patients, who required immediate laparotomy [23]. Moreover, extended or repeated ETN could lead to iatrogenic injuries to the common bile duct or pancreas, with subsequent biliary or pancreatic fistula. In our case, a biliary leakage became evident in the third ETN, suggesting biliary fistulization with the WOPN. Biliary fistula secondary to acute pancreatitis has been rarely reported, and its pathogenesis could be related to the peri-pancreatic necrotic erosion of the common bile duct as well as direct necrosis or autodigestion of the extrahepatic biliary system, or possible iatrogenic injury during necrosectomy [31–33].

In our case, biliary fistula was not evident on CT scan since classical signs such as air into the biliary tree or interruption of choledocal wall opening into the WOPN were not visible; however, the appearance of bile in the WOPN cavity during necrosectomy may be considered a direct sign of biliary fistula [32]. ERCP confirmed the presence of biliary fistula involving distal common bile duct. Management options include primary reconstruction of bile duct with or without Kehr tube, biliary diversion, and in extremis, pancreaticoduodenectomy [9, 32–34]. Since the biliary lesion appeared to be small, a stenting was decided with resolution of the leak. Bilio-pancreatic endoscopy and stenting still has not a clear role in severe necrotizing pancreatitis complicated by biliary fistula, while its role in case of fistulization to pseudocyst is much more practiced [35, 36].

Our case suggests that the evident advantages of minimally invasive techniques should be always cautiously considered together with their potential drawbacks. Maybe treating our patient with delayed open necrosectomy without ETN would have spared a biliary complication to the patient, shortening the clinical course with a better clinical outcome. A priori exclusion of open necrosectomy, if indicated, should be discouraged. Moreover, a delayed VARD was necessary due to ongoing sepsis and extended WOPN after several weeks from admission, probably because endoscopic necrosectomy was not sufficient to achieve an adequate local control of the WOPN. Therefore, a single minimally invasive technique could be inadequate to provide a resolution in such cases of necrotizing pancreatitis. A persistent repetition of a single treatment, even if minimally invasive, could lead to further complications beyond those directly related to the disease itself and should be discouraged.

## Conclusions

To the best of our knowledge, the case presented is the first one of severe necrotizing pancreatitis complicated by biliary fistula treated by a combination of ETN, PD, VARD, and ERCP with biliary stenting. Delay of any procedure after 4–6 weeks from the onset of pancreatitis could be helpful for patient stabilization. ETN, VARD, and PD should be tailored on localization (medial vs. lateral, retroperitoneal vs. intraperitoneal) and on quality (mostly fluid vs. mostly solid) of the collections. A combination of different approaches, even including open necrosectomy when indicated, could significantly optimize the clinical management in critically ill patients affected by complicated necrotizing pancreatitis. Recent literature supports that mini-invasive approaches are associated with better outcomes over early open necrosectomy. However, minimally invasive techniques are not free from complications, and surgical necrosectomy should not be excluded a priori and could still have an indication in some cases of necrotizing pancreatitis.

## Abbreviations

ARDS: Acute respiratory distress syndrome
CVVH: Continuous veno-venous hemofiltration
ERCP: Endoscopic retrograde cholangiopancreatography
ETN: Endoscopic transgastric necrosectomy
MOF: Multi-organ failure
PD: Percutaneous drainage
VARD: Video-assisted retroperitoneal debridement
WOPN: Walled-off pancreatic necrosis
